# Prevalence of Gestational Diabetes in India by Individual Socioeconomic, Demographic, and Clinical Factors

**DOI:** 10.1001/jamanetworkopen.2020.25074

**Published:** 2020-11-09

**Authors:** Goutham Swaminathan, Akshay Swaminathan, Daniel J. Corsi

**Affiliations:** 1 Goergen Institute for Data Science and University of Rochester, Rochester, New York; 2 Quantitative Sciences, Flatiron Health, New York, New York; 3 Ottawa Hospital Research Institute, Ottawa, Ontario, Canada; 4 University of Ottawa, Ottawa, Ontario, Canada

## Abstract

**Importance:**

Gestational diabetes is common in pregnancy and is associated with adverse pregnancy and fetal outcomes. Currently, population-based data on the prevalence of gestational diabetes are limited in India.

**Objective:**

To provide a comprehensive national assessment of gestational diabetes in India and its socioeconomic, demographic, and geographic associations, using elevated random blood glucose data as a proxy for a gestational diabetes diagnosis.

**Design, Setting, and Participants:**

This cross-sectional study analyzed the fourth National Family Health Survey, conducted in India between January 2015 and December 2016. This nationally representative sample comprised 699 686 women 15 to 49 years of age, of whom 32 428 (4.6%) were pregnant. Data were analyzed between July and December 2019 and between July and August 2020.

**Exposures:**

Age, body mass index, hypertension, wealth, and social caste were factors potentially associated with gestational diabetes.

**Main Outcomes and Measures:**

Gestational diabetes, defined as elevated random blood glucose according to predetermined thresholds (≥200 mg/dL for nonfasting, ≥92 mg/dL for fasting).

**Results:**

Of the 31 746 pregnant women with complete data in the study, the mean (SD) age was 24.3 (4.7) years, and the mean (SD) gestational age was 5.1 (2.3) months. The weighted age-adjusted prevalence of gestational diabetes was 1.3% (95% CI, 1.1%-1.5%). The prevalence of gestational diabetes increased with age, from 1.0% (95% CI, 0.5%-1.5%) at age 15 to 19 years to 2.4% (95% CI, 1.0%-3.8%) at age 35 years or older. The age-adjusted prevalence of gestational diabetes was higher among women with a body mass index of 27.5 or greater (1.8%; 95% CI, 1.0%-2.5%) compared with women with a body mass index of less than 18.5 (0.8%; 95% CI, 0.5%-1.1%), among women in the highest wealth quartile (1.7%; 95% CI, 1.1%-2.5%) compared with those in the lowest (0.9%; 95% CI, 0.7%-1.2%), and women in the south (eg, Kerala: 4.5%; 95% CI, 2.4%-6.7%; Telangana: 5.4%; 95% CI, 0.0%-11.0%) compared with the northeast (eg, Assam: 0.23%; 95% CI, 0.0%-0.48%; Mizoram: 0.16%; 95% CI, 0.0%-0.49%).

**Conclusions and Relevance:**

In this study, considerable variation was found in the prevalence of gestational diabetes by state, socioeconomic status, and demographic factors. This finding has implications for the method of gestational diabetes screening in low-resource settings in India, especially in areas or among demographic groups with lower prevalence.

## Key Points

**Question:**

What is the overall prevalence of gestational diabetes in India, and what socioeconomic, demographic, clinical, and geographic factors are associated with it?

**Findings:**

In this cross-sectional study using biometric data from 32 428 women aged 15 to 49 years, there was considerable variation in the prevalence of gestational diabetes across states and by individual socioeconomic, demographic, and clinical factors.

**Meaning:**

These findings emphasize that within the context of universal screening for gestational diabetes in India, tailoring screening methods and potential interventions based on the burden of disease could maximize cost-effectiveness and improve outcomes.

## Teaser

This cross-sectional study provides a comprehensive national assessment of gestational diabetes in India and its socioeconomic, demographic, and geographic associations.

## Introduction

Gestational diabetes (GD) is glucose intolerance that occurs in pregnant women and is characterized by onset or detection during pregnancy.^1^ The clinical effects of GD can range from asymptomatic to severe hyperglycemia.^2^ GD is thought to arise as the result of insulin resistance due to pregnancy hormones, which is not adequately compensated for by the pancreatic β-cells through increased proliferation and insulin secretion.^3^ The entire pathogenesis of the disease remains unknown, although a genetic predisposition is likely due to familial clustering and the identification of several candidate genes associated with increased risk.^4^ Nongenetic factors, including maternal age,^5^ obesity,^6^ diet, and lifestyle, are also associated with GD.^7^

Data from high-income countries indicate that GD complicates approximately 5% to 7% of pregnancies.^8,9^ GD is a global health concern, and in India, the condition affects as many as 5 million women annually.^10^ Recent studies suggest that the incidence of GD has increased in the past decade and that rates may be higher in specific ethnic or racial subgroups.^11,12,13^ GD poses risks for both the mother and fetus. GD is associated with an increased risk of obstetrical complications and adverse fetal outcomes. These include preeclampsia,^14^ Cesarean delivery,^15^ stillbirth,^9^ macrosomia,^14^ and hypoglycemia.^16^ Also, history of GD is associated with elevated risk of GD in future pregnancies and the development of type 2 diabetes^17^ and cardiovascular disease in later life.^18^

There is wide variability in reported prevalence estimates for GD in India, varying from less than 4% to nearly 18%.^19,20^ Despite a government mandate to screen all pregnant women for GD, to date there has been incomplete implementation and uptake of screening programs.^21^ Existing studies on GD in India are limited to single-center studies, with most conducted in urban, hospital-based populations.^19,20,22,23,24^ National data are emerging on the overall prevalence of type 2 diabetes in the Indian population.^25,26^ The overall prevalence is approximately 7%, higher in urban vs rural areas, among older age groups, and among higher socioeconomic status (SES) groups.^27^ Concern exists over an anticipated increase in the prevalence of diabetes and a corresponding rise in GD.^22^ However, to our knowledge, there has been no comprehensive national assessment of GD and socioeconomic, demographic, and geographic factors associated with it.

Furthermore, the social conditions and prevalence of risk factors, such as obesity, hypertension, and type 2 diabetes, are shown to vary substantially across India’s states.^26^ In this study, we investigate the prevalence, socioeconomic, demographic, and health-related factors associated with GD among pregnant women in India using a large-scale nationally representative sample, with elevated random blood glucose as a proxy for GD. Specifically, we investigated the association of SES with GD through the SES markers of social caste, household wealth, and education. We investigated the geographic distribution of GD across states and urban and rural areas.

## Methods

This study was reviewed by the research ethics boards of the Ottawa Health Science Network and the Children’s Hospital of Eastern Ontario. Informed consent was obtained from participants at the time of the survey. We followed the Strengthening the Reporting of Observational Studies in Epidemiology (STROBE) reporting guideline.

### Data Source

Data are from the fourth round of the National Family Health Survey (NFHS-4), conducted between January 20, 2015, and December 4, 2016, by the Ministry of Health and Family Welfare. The NFHS is the Demographic Health Survey for the country of India.^28^ The fourth round collected information from 601 509 households on important population, nutrition, socioeconomic, and maternal-child health indicators. NFHS-4 was the first round to cover all 36 states and union territories in India and included biomarker sampling for height, weight, blood pressure, and blood glucose. The target population of this survey was women 15 to 49 years of age.

### Survey Design

The NFHS-4 used a 2-stage sampling design, stratified by urban and rural areas, to select a representative sample of households.^29^ The NFHS-4 included the population of urban slums in 8 major cities (ie, Chennai, Delhi, Hyderabad, Indore, Kolkata, Meerut, Mumbai, and Nagpur). In the first stage of selection, primary sampling units (PSUs), defined as villages in rural areas and census enumeration blocks in urban areas, were selected. Prior to selection, additional stratification was conducted in rural areas based on the proportion of scheduled castes and tribes within PSUs. PSUs were selected using probability proportional to the population size sampling, and within selected PSUs, field teams carried out a household listing operation.^30^ In the second stage, 22 households per PSU were selected from the newly created household listing using systematic sampling. At the time of the survey, no replacements or changes to selected households were allowed to prevent bias.

Interviewers conducted in-person surveys with respondents in their local language. Interviewers recorded questionnaire data electronically using CAPI software (IdSurvey) to maintain the quality of the data. At the time of the survey, trained health investigators conducted biomarker sampling and measurements of weight, height, blood pressure, and random blood glucose level for respondents. The response rate for NFHS-4 was approximately 98% for households and 97% for eligible women.

### Outcomes

The primary outcome was elevated blood glucose in pregnancy as a proxy for GD. The random blood glucose level of survey respondents was measured using a finger-stick capillary blood specimen and the FreeStyle Optium H glucometer (Right Med Bio System) with glucose test strips. A conversion of whole blood glucose to plasma glucose was applied during data processing, and we use glucose values as given in the data set with no additional adjustment. We used cutoffs to classify GD based on a random glucose test of greater than or equal to 200 mg/dL if the respondent was not fasting and greater than or equal to 92 mg/dL if the respondent reported fasting for 8 or more hours before the test (to convert glucose to micromoles per liter, multiply by 0.0555).^31^ We conducted sensitivity analyses using 160 mg/dL as an alternate cutoff and examined the random glucose values as a continuous variable.

### Study Variables and Factors Associated With Gestational Diabetes

We examined respondent characteristics for age, trimester of pregnancy, parity, current smoking, current alcohol use, and urban or rural place of residence. Parity was based on the number of children ever born and categorized as 0, 1, or 2 or more. Smoking and alcohol were self-reported. The urban or rural location corresponds to the census definition. We considered social caste, household wealth, and education as markers of SES. Household wealth is an index based on indicators of asset ownership and housing characteristics reported in the survey.^32^ This index has been developed and validated in several countries and is consistent with measures of income and expenditure.^33^ The measure includes housing materials, the type of water and sanitation facilities, and assets (eg, ownership of the home, car, computer, mobile phone), which are weighted and combined using a principal component analysis procedure.^34^ Four categories of wealth, from most deprived to least deprived, were created using national level quintiles.^35^ Education was categorized in 4 levels by the number of years completed (no education, primary, secondary, and higher secondary or college). Social caste is based on self-reports and categorized as general caste, scheduled caste, scheduled tribe, other backward classes (OBC), and no caste. In India, the OBC is a large and heterogeneous population group, whereas scheduled castes and scheduled tribes are considered lower status and are socially marginalized.^36^ Health-related risk factors included hypertension and body mass index (BMI; calculated as weight in kilograms divided by height in meters squared). We averaged the 3 systolic and the 3 diastolic blood pressure measurements obtained from survey participants. The cutoff^37^ for hypertension was systolic blood pressure greater than or equal to 140 mm Hg or diastolic blood pressure greater than or equal to 90 mm Hg. Blood pressure readings were taken from the same arm with at least 5 minutes between each measurement and 5 minutes of sitting before the first measurement. Also, individuals who reported taking blood pressure medication were considered hypertensive. BMI was calculated for survey respondents with accurate height and weight measurements. We categorized BMI into 4 groups: less than 18.5, 18.5 to 22.9, 23.0 to 27.4, and 27.5 or higher.^38^

### Statistical Analysis

We calculated the prevalence of GD across categories of the study variables, accounting for the survey design characteristics and sampling weights. We used logistic regression to assess associations between study variables and GD. Regression models accounted for survey design characteristics, sampling weights, and state fixed effects. Union territories were combined in regression models due to the smaller sample sizes. Associations are presented as unadjusted and adjusted odds ratios (ORs) and 95% CIs, all hypothesis tests were 2-sided, and the criterion for statistical significance was set at α = .05. We conducted subgroup analyses by states and regions and tests of statistical interaction were performed using 2-sided Wald tests. The most influential risk factors based on statistical significance and coefficient magnitude were selected to generate an additive risk model for the cumulative probability of GD when considering multiple factors simultaneously. We conducted region-specific analyses across the 6 primary geographic regions: south, north, east, northeast, west, and central. All analyses were done in the R version 3.5.2 (R Project for Statistical Computing) and the survey package.

## Results

A total of 699 686 women participated in the NFHS-4 survey. From this population, we defined a sample of 32 428 women (4.6%) who were pregnant at the time of the survey. After excluding respondents with missing data on outcomes or covariates (682 [2.1%]), the final analytical sample size was 31 746 women (Table 1). The mean (SD) age of women was 24.3 (4.7) years, and the mean (SD) gestational age was 5.1 (2.3) months (Table 2). Overall, 373 women (1.2%) in the sample had glucose in pregnancy of 200 mg/dL or greater, and 609 women (1.9%) had glucose of 160 mg/dL or greater. Descriptive analyses of the alternate definition of GD based on a cutoff of random glucose of 160 mg/dL or greater are given in eTable 1 and eTable 2 in the Supplement. The overall prevalence of GD was 1.3% (95% CI, 1.1%-1.6%), and the age-adjusted prevalence was 1.3% (95% CI, 1.1%-1.5%). The prevalence of GD increased with age, from 1.0% (95% CI, 0.5%-1.5%) among women aged 15 to 19 years to 2.4% (95% CI, 1.0%-3.8%) among women aged 35 years and older. Elevated glucose was relatively stable across trimesters of pregnancy, varying between 1.5% (95% CI, 1.0%-1.9%) among women in the first trimester and 1.6% (95% CI, 1.1%-2.1%) among women in the third trimester, but somewhat lower among women in the second trimester (1.0%; 95% CI, 0.8%-1.3%). A correlation matrix between continuous demographic and clinical characteristics is in eFigure 1 in the Supplement.

**Table 1.** Characteristics of the Study Population and Prevalence of GD, India, 2015-2016^a^

Characteristics	All participants, No. (%)	Gestational diabetes prevalence		
		Women with GD, No. (%)	% (95% CI)	Age-adjusted % (95% CI)
All India	31 746 (100)	373 (100)	1.3 (1.1-1.6)	1.3 (1.1-1.5)
Age, y				
15-19	3589 (12.7)	29 (9.2)	1.0 (0.5-1.5)	NA
20-24	13 472 (44.3)	137 (36.0)	1.1 (0.8-1.4)	NA
25-29	9614 (29.9)	116 (35.4)	1.6 (1.1-2.1)	NA
30-34	3564 (9.6)	68 (13.0)	1.8 (1.2-2.4)	NA
≥35	1507 (3.6)	23 (6.5)	2.4 (1.0-3.8)	NA
Parity, excluding index pregnancy				
0	12 336 (40.6)	146 (38.5)	1.3 (0.9-1.7)	1.4 (1.0-1.9)
1	9841 (31.8)	100 (26.8)	1.1 (0.8-1.5)	1.1 (0.8-1.4)
≥2	9569 (27.7)	127 (33.8)	1.7 (1.2-2.1)	1.3 (0.8-1.9)
Trimester of pregnancy				
First	9454 (29.6)	121 (32.4)	1.5 (1.0-1.9)	1.4 (1.0-1.9)
Second	12 687 (40.0)	125 (30.9)	1.0 (0.8-1.3)	1.0 (0.8-1.2)
Third	9598 (30.4)	126 (35.9)	1.6 (1.1-2.1)	1.5 (1.1-2.0)
Current smoking	146 (0.2)	1 (0.1)	0.5 (0.0-1.7)	0.4 (0.0-1.1)
Current alcohol use	585 (0.8)	11 (1.4)	2.3 (0.0-4.7)	2.1 (0.2-3.9)
Wealth				
1, Lowest	7981 (24.7)	75 (18.0)	1.0 (0.7-1.2)	0.9 (0.7-1.2)
2	7973 (23.3)	79 (20.4)	1.2 (0.7-1.6)	1.2 (0.8-1.6)
3	7946 (25.5)	107 (26.4)	1.4 (1.0-1.8)	1.4 (1.0-1.8)
4, Highest	7846 (26.5)	112 (35.3)	1.8 (1.2-2.4)	1.7 (1.1-2.3)
Education				
No schooling	8050 (24.9)	106 (26.0)	1.4 (1.0-1.8)	1.3 (0.9-1.6)
Primary or middle	10 004 (30.9)	104 (27.8)	1.2 (0.9-1.5)	1.2 (0.9-1.5)
Secondary	6639 (20.7)	68 (20.8)	1.3 (0.8-1.9)	1.4 (0.8-2.0)
≥Senior secondary	7053 (23.6)	95 (25.4)	1.4 (0.9-2.0)	1.4 (0.9-1.9)
Social caste				
General caste	5518 (19.0)	68 (21.4)	1.5 (0.9-2.1)	1.4 (0.8-2.0)
Scheduled caste	5996 (21.5)	51 (13.7)	0.9 (0.6-1.2)	0.8 (0.5-1.1)
Scheduled tribe	6275 (10.2)	68 (10.4)	1.4 (0.7-2.0)	1.3 (0.7-1.9)
Other backward class	12 684 (45.2)	172 (49.3)	1.5 (1.1-1.8)	1.4 (1.1-1.8)
No caste	1273 (4.1)	14 (5.2)	1.7 (0.4-3.1)	1.7 (0.5-3.0)
Body mass index^b^				
<18.5	4192 (14.0)	42 (8.7)	0.8 (0.5-1.1)	0.8 (0.5-1.1)
18.5-23	17 633 (54.8)	184 (49.4)	1.2 (0.9-1.5)	1.2 (0.9-1.5)
23-27.5	7818 (24.1)	104 (31.3)	1.7 (1.1-2.3)	1.6 (1.1-2.2)
>27.5	2103 (7.1)	43 (10.6)	2.0 (1.1-2.9)	1.8 (1.0-2.5)
Hypertension	1662 (4.4)	31 (6.3)	1.9 (1.0-2.8)	1.7 (0.9-2.5)
Urban residence	7555 (27.8)	117 (36.7)	1.8 (1.2-2.4)	1.7 (1.1-2.3)

Abbreviations: GD, gestational diabetes; NA, not applicable.

^a^ GD was defined as random glucose level of 200 mg/dL or greater (to convert to micromoles per liter, multiply by 0.0555).

^b^ Body mass index calculated as weight in kilograms divided by height in meters squared.

**Table 2.** Demographic and Clinical Characteristics of the Study Population and Women with Gestational Diabetes, India, 2015-2016

Characteristic	Total (N = 31 746)		Gestational diabetes (n = 373)		
	Mean (SD)	Median (IQR)	Mean (SD)	Age-adjusted mean	Median (IQR)
Age, y	24.3 (4.7)	24.0 (21.0-27.0)	25.5 (5.1)	25.5	25.0 (22.0-28.0)
Body mass index^a^	21.9 (3.6	21.4 (19.6-23.7)	22.7 (3.9)	22.7	22.1 (19.8-24.8)
Systolic BP, mm Hg	108.3 (11.5)	107.3 (100.3-115.7)	111.2 (13.1)	111.2	111.3 (102.3-118.0)
Diastolic BP, mm Hg	71.5 (8.9)	71.0 (65.7-77.0)	73.3 (9.7)	73.3	73.3 (67.7-78.0)
Glucose, g/dL	95.5 (20.4)	93.0 (81.0-106.0)	117.2 (49.6)	117.2	102.0 (96.0-118.0)
Gestational age, mo	5.1 (2.3)	5.0 (3.0-7.0)	5.1 (2.4)	5.1	5.0 (3.0-7.0)

Abbreviations: BP, blood pressure; IQR, interquartile range.

SI conversion factor: To convert glucose to micrograms per liter, multiply by 0.0555.

^a^ Body mass index calculated as weight in kilograms divided by height in meters squared.

The age-adjusted prevalence of GD was higher in urban areas compared with rural areas (1.7% [95% CI, 1.1%-2.3%] vs 1.2% [95% CI, 0.9%-1.4%]), although this was not statistically significant (*P* = .05). Women with a BMI greater than 27.5 had a higher age-adjusted prevalence of GD than women with a BMI less than 18.5 (1.8% [95% CI, 1.0%-2.5%] vs 0.8% [95% CI, 0.5%-1.1%]). Women who had no caste and women who had higher secondary education or more had the highest age-adjusted prevalence of GD within their respective categories (1.7% [95% CI, 0.5%-3.0%] and 1.4% [95% CI, 0.9%-1.9%], respectively). Women who were in the highest wealth quartile had the highest prevalence of GD (1.7% [95% CI, 1.1%-2.3%]) compared with the other wealth classes (first quartile: 0.9% [95% CI, 0.7%-1.2%]; second: 1.2% [95% CI, 0.8%-1.6%]; third: 1.4% [95% CI, 1.0%-1.8%]).

### Multivariable Analyses of GD Risk Factors

A multivariable logistic regression model indicated that age was the strongest factor associated with GD, followed by BMI, household wealth, caste, and hypertension (Table 3). The OR for GD was 2.37 (95% CI, 1.01-5.60) for women aged 35 years and older compared with women aged 15 to 19 years. Among women with a BMI of greater than 27.5, the OR for GD was 1.74 (95% CI, 0.97-3.12) compared with women with a BMI of less than 18.5. Compared with women who were members of scheduled castes, women who were members of scheduled tribes had an OR for GD of 2.35 (95% CI, 1.24-4.47). Wealth was positively associated with GD, and the OR was 2.27 (95% CI, 1.27-4.04) for households in the highest wealth quartile compared with the lowest after accounting for covariates. Hypertension in pregnancy and residence in an urban area were not statistically significantly associated with GD in adjusted analyses. Analyses of GD using a cutoff of 160 mg/dL and continuous random glucose values revealed similar associations with risk factors (eTable 3 and eTable 4 in the Supplement). Age, wealth, BMI, and caste emerged as the factors more strongly associated with blood glucose in the multivariable adjusted model.

**Table 3.** Odds Ratios From Logistic Regression Analysis of the Association Between Maternal Demographic and Clinical Characteristics and Risk of Gestational Diabetes, India, 2015-2016

Characteristic	Odds ratio (95% CI)	
	Unadjusted	Mutually adjusted
Age, y		
15-19	1.00 (1.00-1.00)	1.00 (1.00-1.00)
20-24	1.12 (0.63-2.01)	1.21 (0.67-2.15)
25-29	1.64 (0.90-2.99)	1.61 (0.81-3.21)
30-34	1.89 (1.04-3.43)	1.82 (0.89-3.74)
≥35	2.51 (1.16-5.40)	2.37 (1.01-5.60)
Parity, excluding index pregnancy		
0	1.00 (1.00-1.00)	1.00 (1.00-1.00)
1	0.89 (0.59-1.34)	0.78 (0.51-1.19)
≥2	1.29 (0.87-1.93)	1.05 (0.54-2.05)
Wealth		
1, Lowest	1.00 (1.00-1.00)	1.00 (1.00-1.00)
2	1.20 (0.77-1.87)	1.49 (0.96-2.30)
3	1.43 (0.97-2.10)	1.90 (1.16-3.10)
4, Highest	1.85 (1.20-2.87)	2.27 (1.27-4.04)
Body mass index^a^		
<18.5	1.00 (1.00-1.00)	1.00 (1.00-1.00)
18.5-23	1.46 (0.96-2.21)	1.39 (0.91-2.12)
23-27.5	2.11 (1.28-3.46)	1.70 (1.04-2.77)
>27.5	2.43 (1.39-4.24)	1.74 (0.97-3.12)
Hypertension	1.45 (0.86-2.44)	1.47 (0.89-2.44)
Education		
No schooling	1.00 (1.00-1.00)	1.00 (1.00-1.00)
Primary or middle	0.86 (0.60-1.24)	0.83 (0.55-1.25)
Secondary	0.96 (0.59-1.56)	0.82 (0.47-1.44)
≥Senior secondary	1.03 (0.65-1.63)	0.62 (0.34-1.14)
Social caste		
General caste	1.79 (1.06-3.01)	1.43 (0.83-2.48)
Scheduled caste	1.00 (1.00-1.00)	1.00 (1.00-1.00)
Scheduled tribe	1.60 (0.90-2.85)	2.35 (1.24-4.47)
Other backward class	1.73 (1.13-2.64)	1.46 (0.96-2.22)
No caste	2.06 (0.91-4.64)	1.57 (0.65-3.78)
Urban residence	1.51 (1.03-2.22)	1.18 (0.82-1.70)

^a^ Body mass index calculated as weight in kilograms divided by height in meters squared.

### Cumulative Effect of Multiple Risk Factors

**Figure 1.**
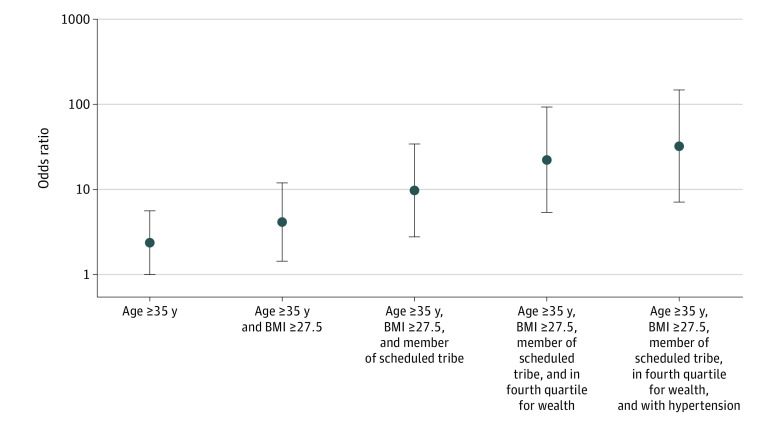
Additive Risk Plot Showing Odds Ratios for Gestational Diabetes When Considering Multiple Risk Factors Simultaneously Gestational diabetes was defined as random glucose level of 200 mg/dL or greater (to convert to micromoles per liter, multiply by 0.0555). BMI, calculated as weight in kilograms divided by height in meters squared, indicates body mass index.

We calculated ORs for risk of GD with multiple simultaneous risk factors, focusing on the most influential risk factors from the mutually adjusted analyses (Figure 1). All reference levels are defined as described earlier. Women 35 years of age or older had an odds ratio of 2.37 (95% CI, 1.01-5.60) for developing GD compared with women aged 15 to 19 years. If these women also have an elevated BMI (>27.5), the OR increased to 4.14 (95% CI, 1.44-11.92). If the women was a member of a scheduled tribe, the OR for GD increased to 9.72 (95% CI, 2.77-34.18). If these women were in the highest wealth quartile, in addition to having the previously listed risk factors, the OR increased to 22.03 (95% CI, 5.34-90.91). Lastly, if the women had hypertension in addition to all other factors, the OR increased to 32.37 (95% CI, 7.09- 147.70). A similar analysis is given using the threshold for GD of 160 mg/dL in eFigure 2 in the Supplement.

### Variation by State

**Figure 2.**
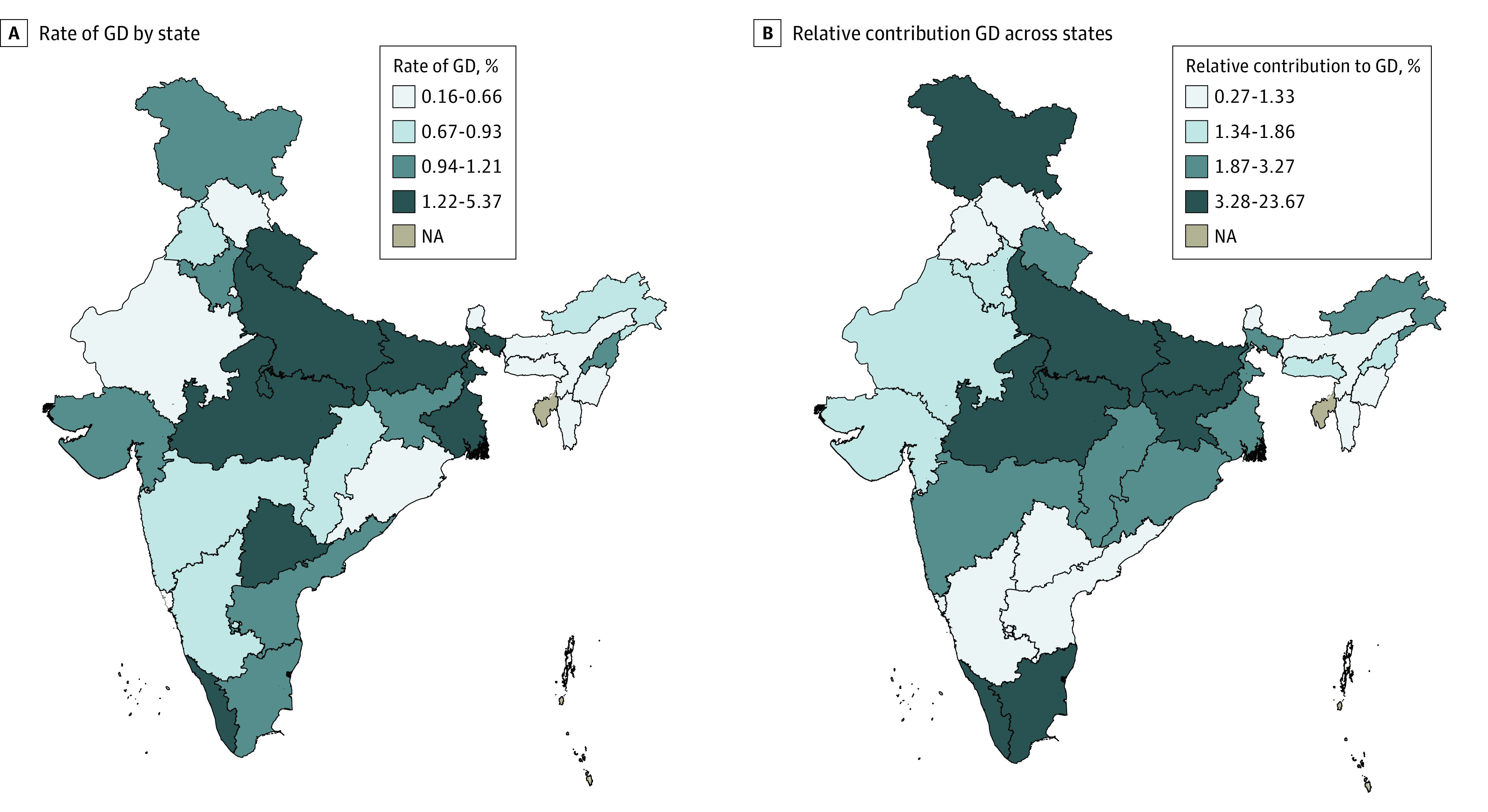
State-Level Age-Adjusted Prevalence of Gestational Diabetes (GD) Among Women Aged 15 to 49 Years and Relative Contribution to the Total Burden of GD by State GD was defined as random glucose level of 200 mg/dL or greater (to convert to micromoles per liter, multiply by 0.0555). NA indicates not applicable.

There was substantial variation in the prevalence of GD across states (*F*_29,17 761_ = 3.33; *P* < .001). At 5.4% (95% CI, 0.0%-11.0%) and 4.5% (95% CI, 2.4%-6.7%), the age-adjusted prevalence of GD was highest in the southern states of Telangana and Kerala, respectively (Figure 2A). The state with the next highest rate of GD was West Bengal in the east, with a rate of 2.3% (95% CI, 0.8%-3.8%). The states of Uttarkhand (central), Bihar (east), Madhya Pradesh (central), and Uttar Pradesh (central) also had rates of GD higher than 1.2% and were in the top quartile. Several states from the north and northeast regions had rates of GD in the lowest quartile, including Meghalya, Rajasthan, Himachal Pradesh, Manipur, Assam, and Mizoram (eg, Assam: 0.23%; 95% CI, 0.0%-0.48%; Mizoram: 0.16%; 95% CI, 0.0%-0.49%). The population distribution of women with GD was concentrated in states in the central and eastern states of Madhya Pradesh, Bihar, and Uttar Pradesh (Figure 2B). Combined, these 3 states contributed 47% of the total cases of GD, followed by 8.8% of cases in Tamil Nadu and Kerala and 5% in Jammu and Kashmir. The state-level prevalence and relative contributions to the total burden of gestational diabetes by state using the cutoff of random glucose level of 160 mg/dL or higher appear in eFigure 3 in the Supplement.

At the state level, the age-adjusted prevalence of type 2 diabetes among women had a correlation of 0.51 with the age-adjusted prevalence of GD, with some variation in the relative ranking of states (eFigure 4 in the Supplement). Using the cutoff of 160 mg/dL, the state-level correlation was 0.61 (eFigure 5 in the Supplement).

### Regional Heterogeneity

We assessed regional heterogeneity in the associations between age, BMI, wealth, hypertension, and GD (eFigure 6 in the Supplement). Age greater than 35 years was a risk factor for GD across all regions, varying from an OR of 1.07 (95% CI, 0.15-7.64) in northeast India to an OR of 4.54 (95% CI, 0.90-22.9) in south India. There was no evidence of effect heterogeneity across regions, indicated by a Wald test (*P* = .57). BMI of 27.5 showed an association that varied between an OR of 0.97 (95% CI, 0.19-4.92) in the west to 3.75 (95% CI, 0.43-32.84) in central India, and the Wald test revealed no heterogeneity in the effect of this risk factor across regions (*P* = .50). Increasing wealth was associated with a higher risk of GD in all regions, although the associations were not statistically significant in some regions, likely due to smaller sample sizes. The Wald test revealed no evidence of effect heterogeneity (*P* = .88). For the association between hypertension and GD, the associations were positive in 5 of 6 regions, although this factor was less statistically robust and failed to reach statistical significance. There was no evidence of heterogeneity across regions (Wald *P* value = .86). Similar results were obtained for gestational diabetes defined by random glucose greater than or equal to 160 mg/dL (eFigure 7 in the Supplement).

## Discussion

This article uses a recent national survey to identify the cross-sectional factors associated with GD in India. We have 3 principal findings. First, we report the national prevalence of gestational diabetes using, for the first time that we are aware of, a consistent methodology and a representative sample with coverage of all states. Rates of GD vary substantially across states, with southern and central states having the highest prevalence. Second, we found fundamental clinical, demographic, and socioeconomic factors associated with GD, including age, hypertension, BMI, social caste, and household wealth. The combined effect of these factors substantially increased the population-level risk of GD. Third, the factors associated with GD were broadly consistent across all regions of India, suggesting the importance of these factors irrespective of geographic location.

The existing literature shows variability regarding estimates of GD prevalence in India, with most studies showing a somewhat higher prevalence than we report here, and varying between 4% to 14%.^19,20,22,23,24^ Differences in the estimated GD prevalence between our study, which reported a prevalence of less than 2%, and other literature may be because of variations in sample sizes, regional variability, and varying cutoffs used for the categorization GD or method of screening. To date, we are not aware of any studies that report nationally representative estimates. Even so, prior studies have reported similar associations between GD and SES and other risk factors, including BMI.

The facotirs associated with GD that we identified are consistent with the findings of previous studies conducted in India and other countries. For example, a study that estimated the prevalence of GD in an urban section of North India found that most women with GD belonged to the middle socioeconomic class.^19^ In contrast, our results showed that most women with GD belonged to the wealthiest socioeconomic class. This disparity may have to do with regional variation as well as with the fact that the previous study was not nationally representative. Also, the study may have used different wealth cutoffs to determine SES categories. Regardless, both studies seem to demonstrate a positive association between SES and GD. Another study examining the association between age and prevalence of GD,^5^ using clinical data from a hospital in Hong Kong, found that the relative risk of GD increased from 2.59 (95% CI, 1.84-3.67) for women aged 25 to 29 years to 10.85 (95% CI, 7.72-15.25) for women aged 35 to 39 years (compared with women aged 20-24 years). Our study also found that the OR of GD for women aged 35 years or older was 2.4 (95% CI, 1.0-5.6), compared with women aged 15 to 19 years, and followed the same trend with age, although the associations were weaker in magnitude.

Some variability was found in the factors associated with GD compared with those associated with type 2 diabetes in India.^26,44^ However, the association between household wealth and GD was similar to type 2 diabetes. The OR for GD for the highest level of wealth was 2.27 (95% CI, 1.27-4.04), compared with 2.31 for the top wealth quintile for type 2 diabetes. The associations between education and GD as well as caste and GD were variable and not consistent with type 2 diabetes. In GD, increasing education was inversely associated with GD, although the association was weak and not statistically reliable. This finding is in contrast to a finding suggesting a positive association between education and type 2 diabetes, although the gradient was less pronounced compared with wealth. Associations between social caste and GD also indicated that scheduled tribes were at elevated risk of GD compared with scheduled castes. In contrast, scheduled tribes had the lowest rates of type 2 diabetes. There was variation in the rates of type 2 diabetes and GD between states, although these 2 measures were moderately correlated. Finally, the urban-rural gradient in GD was weaker compared with type 2 diabetes, and the association was null after accounting for age and other factors.

Our results suggest that gestational diabetes may affect between 5 and 8 million pregnant women in India annually, similar to previous estimates.^10^ Previously, a comprehensive national prevalence estimate of GD did not exist. The NFHS-4 represents an essential milestone for GD research because it was the first survey to record biomarker and socioeconomic data for all 36 states and union territories in India. Prior studies have shown an association between SES indicators and diabetes in the overall population,^26,44^ and this study has allowed us to confirm associations for GD as well.

### Limitations

This report comes from an extensive national survey that has certain limitations. First, the nature of assessing diabetes in this survey limits our ability to distinguish between type 1 diabetes, type 2 diabetes, or GD. We limited the study population to women who were pregnant at the time of the survey and assumed that elevated glucose based on our cutoffs represented GD. However, a proportion of these cases may represent undiagnosed preexisting type 1 or type 2 diabetes.^16^ Second, although no specific guidelines exist for assessing GD status in population-based and epidemiological surveys, there has been much debate regarding the optimal cutoffs to be used for screening and diagnosis of GD and the implications for pregnancy and neonatal outcomes.^39,40,41,42^ As part of The Women in India GD strategy, different nonfasting venous plasma glucose cutoffs following a 75-g oral glucose tolerance test were compared with the glucose values criteria established by the International Association of Diabetes and Pregnancy Study Groups^31^ and the World Health Organization 1999 criteria.^43^ Based on these findings, it was suggested that a cutoff of 140 mg/dL, proposed by the Diabetes in Pregnancy Study Group of India, not be used because of low sensitivity and specificity vs the established cutoffs. Based on this, we determined the outcome of GD using a conservative estimate of 200 mg/dL in nonfasting random glucose, with a sensitivity analyses using 160 mg/dL, given the limitations in establishing a diagnosis of GD using only a random glucose test. Furthermore, the International Association of Diabetes and Pregnancy Study Group recommends screening for GD at 24 to 28 weeks’ gestation, and there may be challenges in screening for GD earlier in pregnancy. In our study population, the mean gestational age was approximately 22 weeks, and we had a roughly even distribution of women across trimesters of pregnancy. Our findings suggested that we were able to capture a similar proportion of women with elevated glucose in each trimester. Using different cutoffs may have led to differential identification of GD cases and have some implications for GD prevalence estimates. However, our analyses using continuous glucose data suggest that similar factors associated with elevated glucose or GD would emerge, regardless of the choice of cutoff value for each state. Finally, this survey is lacking fasting glucose and symptom data, which could be used to make a more definitive diagnosis of GD, although a small minority of women were fasting at the time of the survey.

## Conclusions

Although the genesis of the GD is largely unknown, our study shows important variability across demographic groups and geography in India. These findings emphasize that a tailored approach to managing GD across a diverse country like India may be needed. For instance, within the context of universal screening for GD in India, tailoring the method of screening based on the burden of disease could maximize cost-effectiveness. Community-based lifestyle interventions could be developed and delivered to populations at risk of GD to improve outcomes.

## Notes

Supplement. **eTable 1. **Characteristics of the Study Population and Prevalence of Gestational Diabetes (Random Glucose ≥160 mg/dL), India, 2015-2016 **eTable 2. **Demographic and Clinical Characteristics of the Study Population and Women with Gestational Diabetes (Random Glucose ≥160 mg/dL), India, 2015-2016 **eTable 3. **Unadjusted and Mutually Adjusted Odds Ratios From Logistic Regression Analysis of the Association Between Maternal Demographic and Clinical Characteristics and Risk of Gestational Diabetes (Random Glucose ≥160 mg/dL), India, 2015-2016 **eTable 4. **Unadjusted and Mutually Adjusted β Coefficients From Linear Regression Analysis of the Association Between Maternal Demographic and Clinical Characteristics and Blood Glucose Values (in g/dL), India, 2015-2016 **eFigure 1. **Correlation Matrix Between Demographic and Clinical Characteristics of the Study Population of Pregnant Women (N = 31 746), India, 2015-2016 **eFigure 2. **Additive Risk Plot Showing Odds Ratios for Gestational Diabetes (Random Glucose ≥160 mg/dL) When Considering Multiple Risk Factors Simultaneously **eFigure 3. **State-Level Prevalence of Gestational Diabetes (Random Glucose ≥160 mg/dL) Among Women Aged 15 to 49 Years and Relative Contribution to the Total Burden of Gestational Diabetes by State **eFigure 4. **State-Level Correlation Between the Age-Adjusted Prevalence of Gestational Diabetes and Type 2 Diabetes Among Women 15 to 49 Years of Age, India, 2015-2016 **eFigure 5. **State-Level Correlation Between the Age-Adjusted Prevalence of Gestational Diabetes (Random Glucose ≥160 mg/dL) and Type 2 Diabetes Among Women 15 to 49 Years of Age, India, 2015-2016 **eFigure 6.** Regional Heterogeneity in Odds Ratios for Risk Factors for Gestational Diabetes **eFigure 7. **Regional Heterogeneity in Odds Ratios for Risk Factors for Gestational Diabetes (Random Glucose ≥160 mg/dL)
