# Supplementary material for: Prevalence of Gestational Diabetes in India by Individual Socioeconomic, Demographic, and Clinical Factors
Source: JAMA Netw Open. 2020 Nov 9;3(11):e2025074. doi: 10.1001/jamanetworkopen.2020.25074 (PMC7653498; doi:10.1001/jamanetworkopen.2020.25074)
Supplement: Supplement. — eTable 1. Characteristics of the Study Population and Prevalence of Gestational Diabetes (Random Glucose ≥160 mg/dL), India, 2015-2016 eTable 2. Demographic and Clinical Characteristics of the Study Population and Women with Gestational Diabetes (Random Glucose ≥160 mg/dL), India, 2015-2016 eTable 3. Unadjusted and Mutually Adjusted Odds Ratios From Logistic Regression Analysis of the Association Between Maternal Demographic and Clinical Characteristics and Risk of Gestational Diabetes (Random Glucose ≥160 mg/dL), India, 2015-2016 eTable 4. Unadjusted and Mutually Adjusted β Coefficients From Linear Regression Analysis of the Association Between Maternal Demographic and Clinical Characteristics and Blood Glucose Values (in g/dL), India, 2015-2016 eFigure 1. Correlation Matrix Between Demographic and Clinical Characteristics of the Study Population of Pregnant Women (N = 31 746), India, 2015-2016 eFigure 2. Additive Risk Plot Showing Odds Ratios for Gestational Diabetes (Random Glucose ≥160 mg/dL) When Considering Multiple Risk Factors Simultaneously eFigure 3. State-Level Prevalence of Gestational Diabetes (Random Glucose ≥160 mg/dL) Among Women Aged 15 to 49 Years and Relative Contribution to the Total Burden of Gestational Diabetes by State eFigure 4. State-Level Correlation Between the Age-Adjusted Prevalence of Gestational Diabetes and Type 2 Diabetes Among Women 15 to 49 Years of Age, India, 2015-2016 eFigure 5. State-Level Correlation Between the Age-Adjusted Prevalence of Gestational Diabetes (Random Glucose ≥160 mg/dL) and Type 2 Diabetes Among Women 15 to 49 Years of Age, India, 2015-2016 eFigure 6. Regional Heterogeneity in Odds Ratios for Risk Factors for Gestational Diabetes eFigure 7. Regional Heterogeneity in Odds Ratios for Risk Factors for Gestational Diabetes (Random Glucose ≥160 mg/dL) [file jamanetwopen-e2025074-s001.pdf]

Supplemental Online Content

Swaminathan G, Swaminathan A, Corsi DJ. Prevalence of gestational diabetes in India, by individual socioeconomic, demographic, and clinical factors. *JAMA Netw Open*. 2020;3(11):e2025074. doi:10.1001/jamanetworkopen.2020.25074

eTable 1. Characteristics of the Study Population and Prevalence of Gestational Diabetes (Random Glucose ≥160 mg/dL), India, 2015-2016
eTable 2. Demographic and Clinical Characteristics of the Study Population and Women with Gestational Diabetes (Random Glucose ≥160 mg/dL), India, 2015-2016
eTable 3. Unadjusted and Mutually Adjusted Odds Ratios From Logistic Regression Analysis of the Association Between Maternal Demographic and Clinical Characteristics and Risk of Gestational Diabetes (Random Glucose ≥160 mg/dL), India, 2015-2016
eTable 4. Unadjusted and Mutually Adjusted β Coefficients From Linear Regression Analysis of the Association Between Maternal Demographic and Clinical Characteristics and Blood Glucose Values (in g/dL), India, 2015-2016
eFigure 1. Correlation Matrix Between Demographic and Clinical Characteristics of the Study Population of Pregnant Women (N = 31 746), India, 2015-2016
eFigure 2. Additive Risk Plot Showing Odds Ratios for Gestational Diabetes (Random Glucose ≥160 mg/dL) When Considering Multiple Risk Factors Simultaneously
eFigure 3. State-Level Prevalence of Gestational Diabetes (Random Glucose ≥160 mg/dL) Among Women Aged 15 to 49 Years and Relative Contribution to the Total Burden of Gestational Diabetes by State
eFigure 4. State-Level Correlation Between the Age-Adjusted Prevalence of Gestational Diabetes and Type 2 Diabetes Among Women 15 to 49 Years of Age, India, 2015-2016
eFigure 5. State-Level Correlation Between the Age-Adjusted Prevalence of Gestational Diabetes (Random Glucose ≥160 mg/dL) and Type 2 Diabetes Among Women 15 to 49 Years of Age, India, 2015-2016
eFigure 6. Regional Heterogeneity in Odds Ratios for Risk Factors for Gestational Diabetes
eFigure 7. Regional Heterogeneity in Odds Ratios for Risk Factors for Gestational Diabetes (Random Glucose ≥160 mg/dL)

This supplemental material has been provided by the authors to give readers additional information about their work.

© 2020 Swaminathan G et al. *JAMA Network Open.*

SUPPLEMENTAL APPENDIX

eTable 1. Characteristics of the Study Population and Prevalence of Gestational Diabetes (Random Glucose ≥160 mg/dL), India, 2015-2016

			**Gestational Diabetes Prevalence**							
**Characteristics**	**n**	**%**	**n**	**%**	**% (95% CI)**			**Age-adjusted % (95% CI)**		
All India	31,746	100.0	609	100.0	2.2	(1.9,	2.4)	2.1	(1.8,	2.4)
Age (years)										
15-19 y	3,589	12.7	49	9.3	1.6	(0.9,	2.2)			
20-24 y	13,472	44.3	224	35.7	1.7	(1.4,	2.1)			
25-29 y	9,614	29.9	192	35.0	2.5	(2.0,	3.1)			
30-34 y	3,564	9.6	100	13.1	2.9	(2.1,	3.8)			
35+ y	1,507	3.6	44	7.0	4.2	(2.3,	6.0)			
Parity (not including index pregnancy)										
0	12,336	40.6	233	38.4	2.1	(1.6,	2.5)	2.3	(1.8,	2.8)
1	9,841	31.8	169	28.9	2.0	(1.5,	2.4)	1.9	(1.5,	2.3)
2 or more	9,569	27.7	207	32.2	2.5	(2.0,	3.0)	2.0	(1.4,	2.6)
Trimester of Pregnancy										
1st	9,454	29.6	183	31.4	2.3	(1.8,	2.8)	2.2	(1.7,	2.8)
2nd	12,687	40.0	236	36.3	2.0	(1.6,	2.3)	1.9	(1.5,	2.2)
3rd	9,598	30.4	189	31.8	2.3	(1.7,	2.8)	2.2	(1.7,	2.7)
Current smokers	146	0.2	3	0.1	0.7	-(0.7,	2.0)	0.5	-(0.2,	1.3)
Current alcohol use	585	0.8	16	1.3	3.6	(1.1,	6.0)	3.2	(0.9,	5.4)
Wealth										
Poorest	7,981	24.7	126	18.4	1.6	(1.2,	2.0)	1.5	(1.2,	1.8)
2	7,973	23.3	130	20.5	1.9	(1.4,	2.4)	1.9	(1.4,	2.4)
3	7,946	25.5	170	25.8	2.2	(1.7,	2.7)	2.2	(1.7,	2.7)
Richest	7,846	26.5	183	35.4	2.9	(2.2,	3.6)	2.7	(2.0,	3.4)
Education										
No schooling	8,050	24.9	168	25.3	2.2	(1.7,	2.6)	1.9	(1.5,	2.3)
Primary/middle	10,004	30.9	168	26.7	1.9	(1.5,	2.3)	1.9	(1.5,	2.3)
Secondary	6,639	20.7	111	19.6	2.0	(1.4,	2.7)	2.1	(1.5,	2.8)
Senior second. or above	7,053	23.6	162	28.5	2.6	(1.9,	3.3)	2.5	(1.9,	3.1)
Social Caste										
General caste	5,518	19.0	113	20.7	2.3	(1.7,	3.0)	2.2	(1.5,	2.9)
Scheduled caste	5,996	21.5	93	16.7	1.7	(1.2,	2.1)	1.6	(1.2,	2.1)
Scheduled tribe	6,275	10.2	115	10.6	2.2	(1.4,	3.0)	2.2	(1.4,	2.9)
Other backward class	12,684	45.2	261	46.8	2.2	(1.8,	2.7)	2.2	(1.8,	2.6)
No caste	1,273	4.1	27	5.3	2.8	(1.1,	4.5)	2.8	(1.2,	4.3)

© 2020 Swaminathan G et al. *JAMA Network Open.*

Body mass index										
<18.5	4,192	14.0	65	9.0	1.4	(1.0,	1.8)	1.4	(1.0,	1.8)
18.5-23	17,633	54.8	306	49.9	2.0	(1.6,	2.3)	2.0	(1.6,	2.3)
23-27.5	7,818	24.1	158	27.3	2.4	(1.8,	3.1)	2.3	(1.7,	2.9)
27.5+	2,103	7.1	80	13.7	4.2	(2.8,	5.5)	3.7	(2.5,	4.9)
Hypertension	1,662	4.4	54	7.1	3.5	(2.2,	4.7)	3.1	(2.0,	4.2)
Urban residence	7,555	27.8	179	34.9	2.7	(2.0,	3.4)	2.6	(1.9,	3.2)

© 2020 Swaminathan G et al. *JAMA Network Open.*

eTable 2. Demographic and Clinical Characteristics of the Study Population and Women with Gestational Diabetes (Random Glucose ≥160 mg/dL), India, 2015-2016

	**Total (N=31,746)**					**Gestational Diabetes (n=609)**					
			**Percentiles**						**Percentiles**		
**Characteristic**	**Mean**	**SD**	**25th**	**Median**	**75th**	**Mean**	**SD**	**Age-adjusted Mean**	**25th**	**Median**	**75th**
Age, years	24.3	4.7	21.0	24.0	27.0	25.5	5.2	25.5	22.0	25.0	28.0
Body mass index, kg/m^2	21.9	3.6	19.6	21.4	23.7	22.8	4.2	22.8	19.8	22.0	25.2
Systolic BP, mmHg	108.3	11.5	100.3	107.3	115.7	111.5	13.5	111.5	102.3	111.3	118.3
Diastolic BP, mmHg	71.5	8.9	65.7	71.0	77.0	73.4	9.8	73.4	67.0	73.0	78.3
Glucose, g/dL	95.5	20.4	81.0	93.0	106.0	138.1	47.8	138.1	99.0	125.0	171.0
Gestational age, months	5.1	2.3	3.0	5.0	7.0	5.0	2.3	5.0	3.0	5.0	7.0

© 2020 Swaminathan G et al. *JAMA Network Open.*

eTable 3. Unadjusted and Mutually Adjusted Odds Ratios From Logistic Regression Analysis of the Association Between Maternal Demographic and Clinical Characteristics and Risk of Gestational Diabetes (Random Glucose ≥160 mg/dL), India, 2015-2016

	**Unadjusted**			**Mutually Adjusted**		
	**OR**	**95% CI**		**OR**	**95% CI**	
Age (years)						
15-19 y	1.00	(1.00 -	1.00)	1.00	(1.00 -	1.00)
20-24 y	1.10	(0.70 -	1.74)	1.11	(0.71 -	1.75)
25-29 y	1.62	(1.01 -	2.59)	1.51	(0.90 -	2.52)
30-34 y	1.89	(1.14 -	3.12)	1.73	(0.98 -	3.03)
35+ y	2.71	(1.49 -	4.94)	2.49	(1.31 -	4.74)
Parity (not including index pregnancy)						
0	1.00	(1.00 -	1.00)	1.00	(1.00 -	1.00)
1	0.96	(0.70 -	1.31)	0.86	(0.62 -	1.19)
2 or more	1.24	(0.92 -	1.67)	1.07	(0.67 -	1.70)
Wealth						
Poorest	1.00	(1.00 -	1.00)	1.00	(1.00 -	1.00)
2	1.18	(0.85 -	1.66)	1.41	(0.99 -	2.00)
3	1.37	(1.01 -	1.86)	1.65	(1.11 -	2.45)
Richest	1.82	(1.31 -	2.54)	1.92	(1.21 -	3.05)
Body mass index						
<18.5	1.00	(1.00 -	1.00)	1.00	(1.00 -	1.00)
18.5-23	1.42	(1.03 -	1.98)	1.38	(0.99 -	1.92)
23-27.5	1.78	(1.20 -	2.64)	1.46	(0.99 -	2.17)
27.5+	3.08	(1.98 -	4.79)	2.23	(1.42 -	3.51)
Hypertension	1.67	(1.13 -	2.46)	1.61	(1.10 -	2.36)
Education						
No schooling	1.00	(1.00 -	1.00)	1.00	(1.00 -	1.00)
Primary/middle	0.85	(0.64 -	1.13)	0.84	(0.62 -	1.14)
Secondary	0.93	(0.64 -	1.34)	0.81	(0.53 -	1.25)
Senior second. or above	1.20	(0.86 -	1.67)	0.81	(0.53 -	1.25)
Social Caste						
General caste	1.41	(0.94 -	2.12)	1.13	(0.72 -	1.77)
Scheduled caste	1.00	(1.00 -	1.00)	1.00	(1.00 -	1.00)
Scheduled tribe	1.34	(0.85 -	2.09)	1.79	(1.09 -	2.95)
Other backward class	1.34	(0.96 -	1.89)	1.20	(0.86 -	1.69)
No caste	1.70	(0.89 -	3.23)	1.36	(0.68 -	2.74)
Urban residence	1.40	(1.04 -	1.88)	1.08	(0.80 -	1.45)

© 2020 Swaminathan G et al. *JAMA Network Open.*

eTable 4. Unadjusted and Mutually Adjusted β Coefficients From Linear Regression Analysis of the Association Between Maternal Demographic and Clinical Characteristics and Blood Glucose Values (in g/dL), India, 2015-2016

	Unadjusted			Mutually Adjusted		
	β	SE	95% CI	β	SE	95% CI
Age (years)						
15-19 y	0.00			0.00		
20-24 y	0.88	0.53	-(0.16 - 1.92)	0.88	0.55	-(0.20 - 1.96)
25-29 y	2.95	0.57	(1.82 - 4.08)	2.94	0.66	(1.65 - 4.24)
30-34 y	4.72	0.73	(3.29 - 6.14)	4.63	0.80	(3.07 - 6.20)
35+ y	5.55	1.23	(3.13 - 7.97)	6.11	1.26	(3.65 - 8.57)
Parity (not including index pregnancy)						
0.00	0.00			0.00		
1.00	-1.23	0.42	-(2.06 - -0.41)	-1.94	0.44	-(2.80 - -1.09)
2 or more	-1.42	0.40	-(2.21 - -0.63)	-2.47	0.50	-(3.45 - -1.49)
Wealth						
Poorest	0.00			0.00		
2.00	0.94	0.43	(0.09 - 1.79)	0.84	0.47	-(0.08 - 1.77)
3.00	1.07	0.45	(0.19 - 1.95)	0.49	0.55	-(0.58 - 1.56)
Richest	5.03	0.52	(4.02 - 6.05)	2.21	0.71	(0.82 - 3.60)
Body mass index						
<18.5	0.00			0.00		
18.5-23	-0.32	0.49	-(1.29 - 0.65)	-0.85	0.48	-(1.80 - 0.09)
23-27.5	1.07	0.56	-(0.03 - 2.16)	-0.58	0.56	-(1.67 - 0.51)
27.5+	5.56	0.97	(3.67 - 7.45)	3.07	0.98	(1.16 - 4.99)
Hypertension	2.33	0.83	(0.69 - 3.96)	1.18	0.81	-(0.41 - 2.78)
Education						
No schooling	0.00			0.00		
Primary/middle	-0.63	0.41	-(1.44 - 0.19)	-1.11	0.42	-(1.94 - -0.29)
Secondary	0.45	0.48	-(0.49 - 1.39)	-0.46	0.52	-(1.48 - 0.56)
Senior second. or above	3.58	0.50	(2.61 - 4.56)	0.38	0.56	-(0.71 - 1.48)
Social Caste						
General caste	2.51	0.57	(1.40 - 3.62)	1.66	0.59	(0.50 - 2.81)
Scheduled caste	0.00			0.00		
Scheduled tribe	1.72	0.60	(0.54 - 2.91)	2.37	0.66	(1.07 - 3.66)
Other backward class	1.16	0.43	(0.31 - 2.00)	0.92	0.43	(0.08 - 1.76)

© 2020 Swaminathan G et al. *JAMA Network Open.*

No caste	3.84	1.32	(1.25 -	6.44)	3.15	1.38	(0.45 -	5.84)
Urban residence	2.03	0.47	(1.11 -	2.95)	0.10	0.50	-(0.88 -	1.07)

© 2020 Swaminathan G et al. *JAMA Network Open.*

eFigure 1. Correlation Matrix Between Demographic and Clinical Characteristics of the Study Population of Pregnant Women (N = 31 746), India, 2015-2016

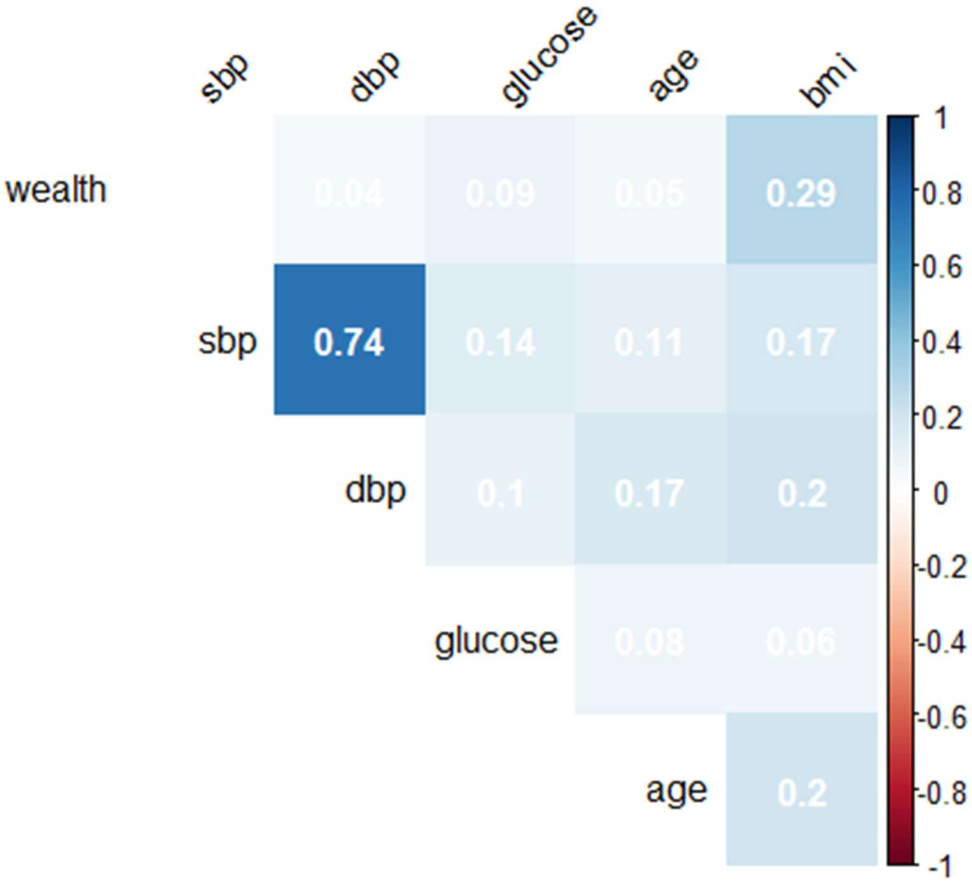

© 2020 Swaminathan G et al. *JAMA Network Open.*

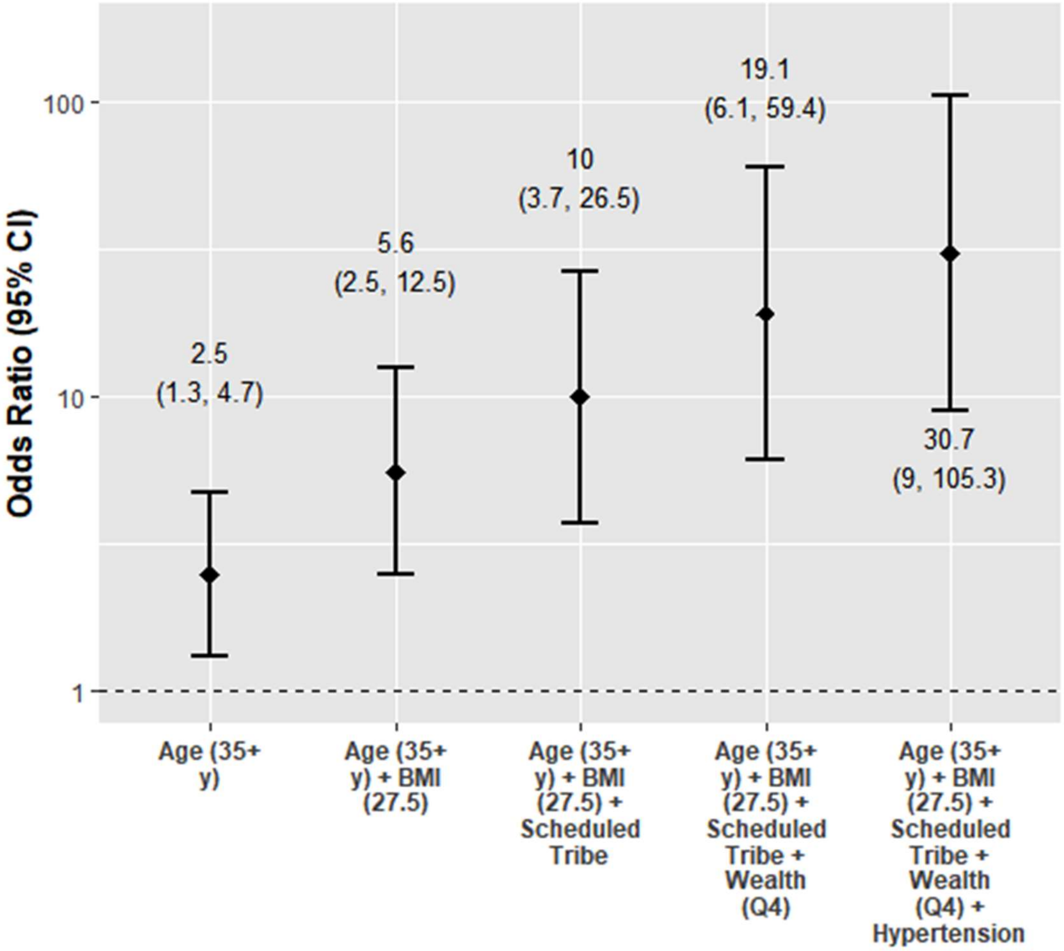

eFigure 2. Additive Risk Plot Showing Odds Ratios for Gestational Diabetes (Random Glucose ≥160 mg/dL) When Considering Multiple Risk Factors Simultaneously

Notes: 1=Age (>35 years), 2=Obesity (BMI>27.5 kg/m^2), 3=Scheduled Tribe, 4=Highest wealth quartile, 5=hypertension

© 2020 Swaminathan G et al. *JAMA Network Open.*

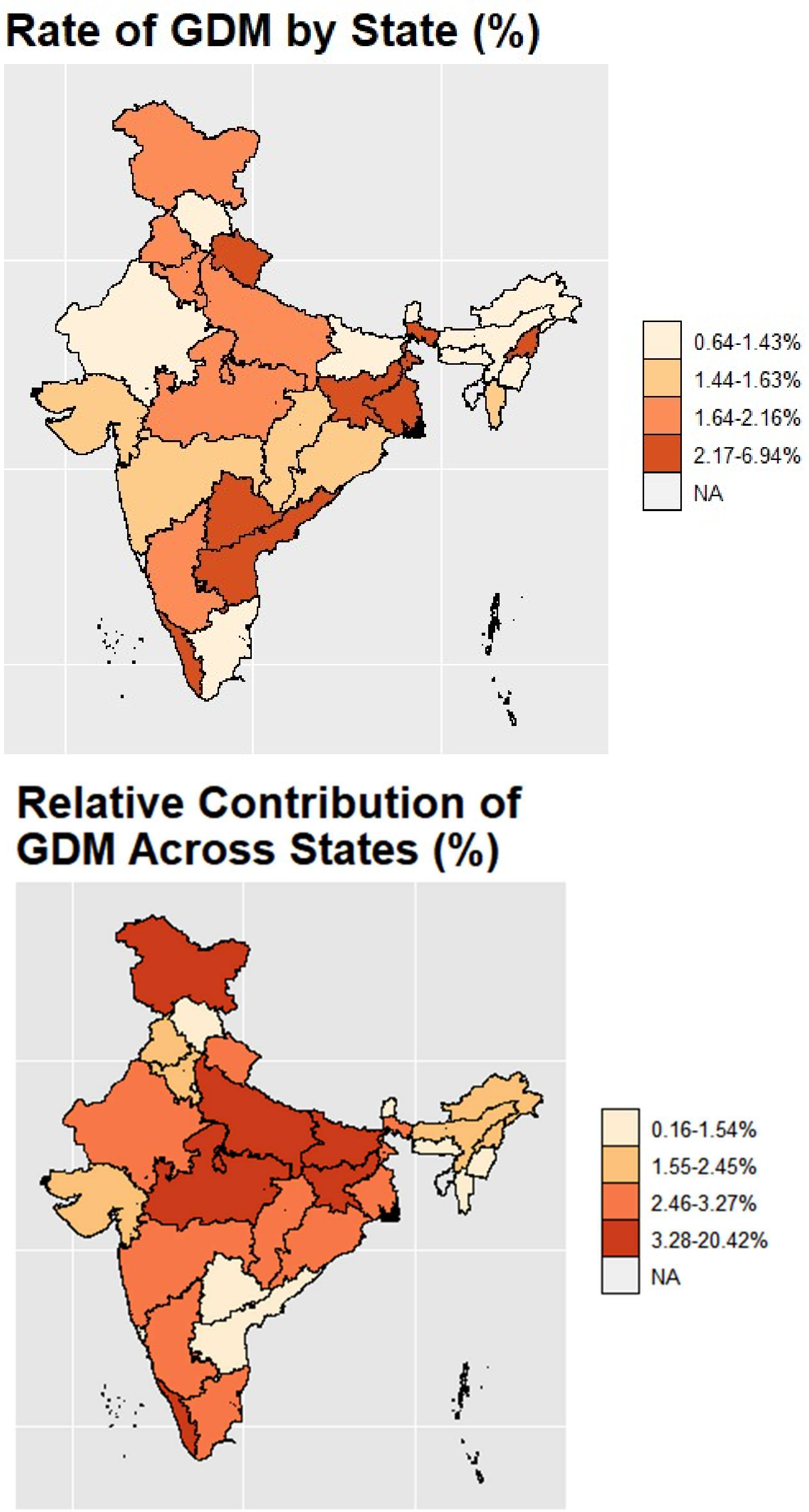

eFigure 3. State-Level Prevalence of Gestational Diabetes (Random Glucose ≥160 mg/dL) Among Women Aged 15 to 49 Years and Relative Contribution to the Total Burden of Gestational Diabetes by State

Darker colours indicate higher prevalence.

© 2020 Swaminathan G et al. *JAMA Network Open.*

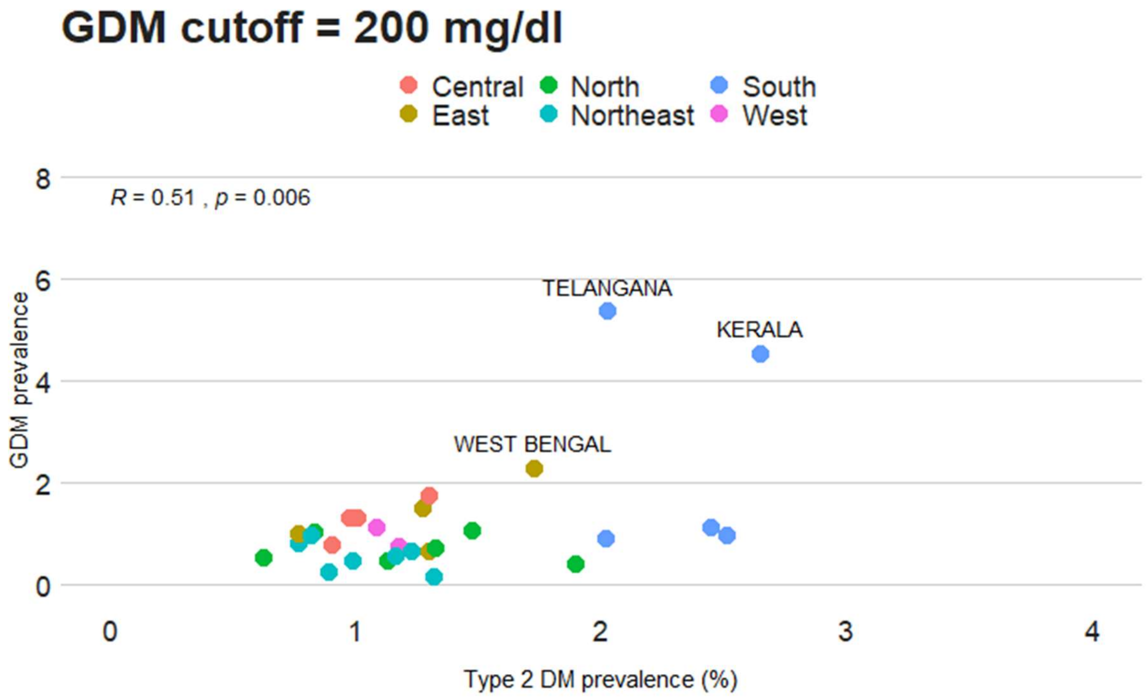

eFigure 4. State-Level Correlation Between the Age-Adjusted Prevalence of Gestational Diabetes and Type 2 Diabetes Among Women 15 to 49 Years of Age, India, 2015-2016

© 2020 Swaminathan G et al. *JAMA Network Open.*

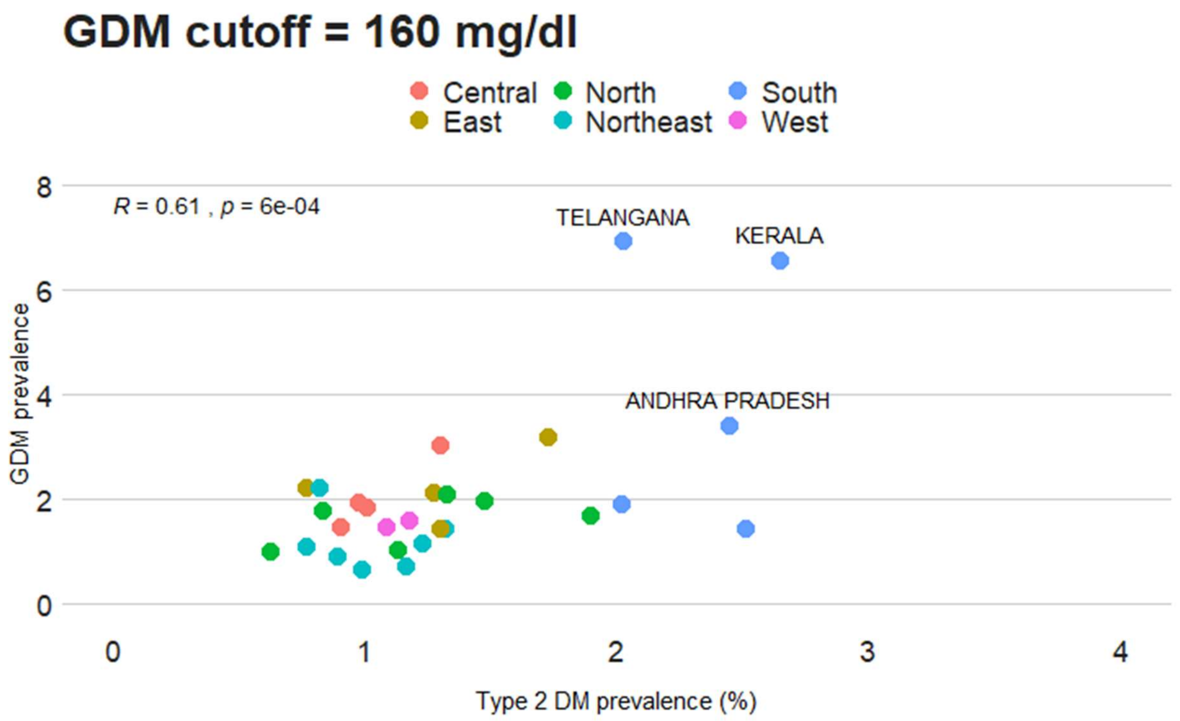

eFigure 5. State-Level Correlation Between the Age-Adjusted Prevalence of Gestational Diabetes (Random Glucose ≥160 mg/dL) and Type 2 Diabetes Among Women 15 to 49 Years of Age, India, 2015-2016

© 2020 Swaminathan G et al. *JAMA Network Open.*

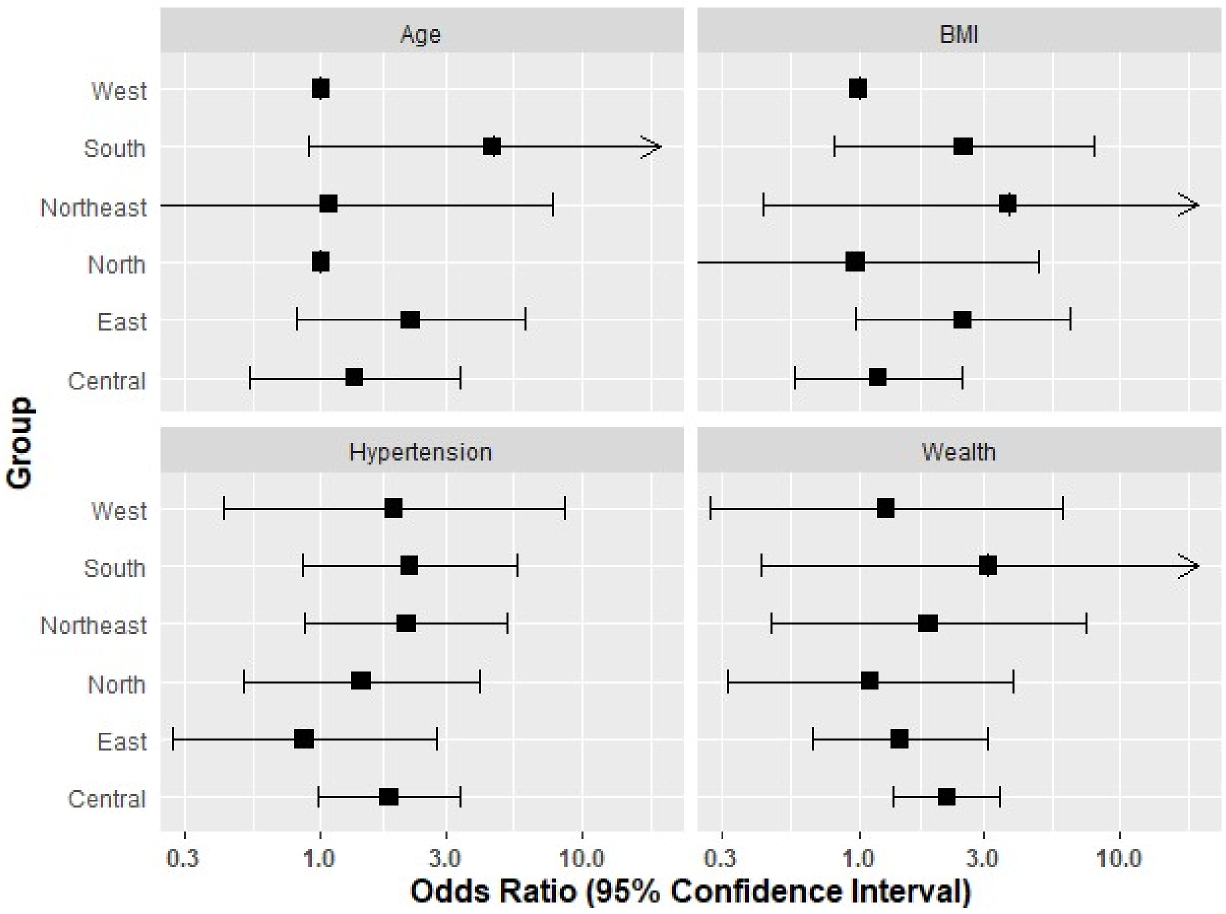

eFigure 6. Regional Heterogeneity in Odds Ratios for Risk Factors for Gestational Diabetes

Age 35+ years, BMI>27.5 kg/m^2, hypertension, and highest wealth quartile

© 2020 Swaminathan G et al. *JAMA Network Open.*

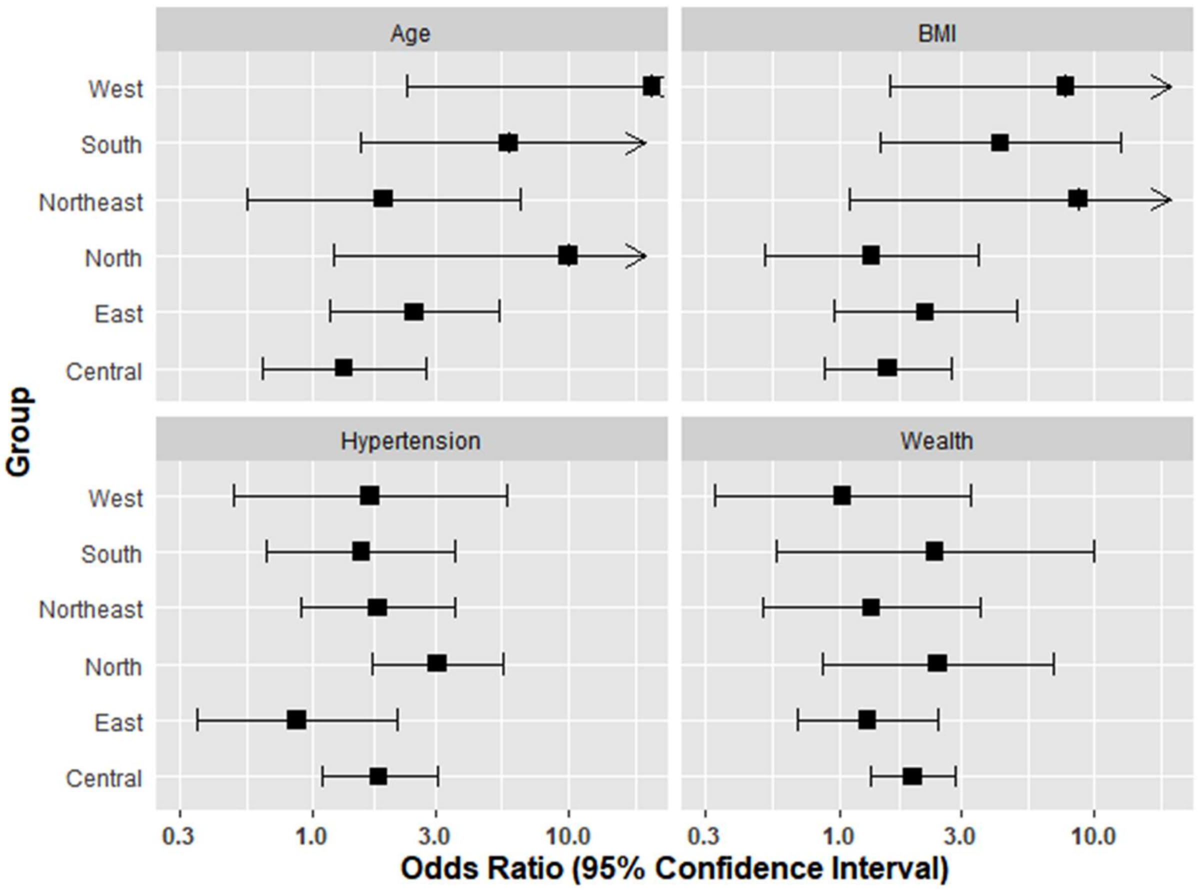

eFigure 7. Regional Heterogeneity in Odds Ratios for Risk Factors for Gestational Diabetes (Random Glucose ≥160 mg/dL)
Age 35+ years, BMI>27.5 kg/m^2, hypertension, and highest wealth quartile

© 2020 Swaminathan G et al. *JAMA Network Open.*
